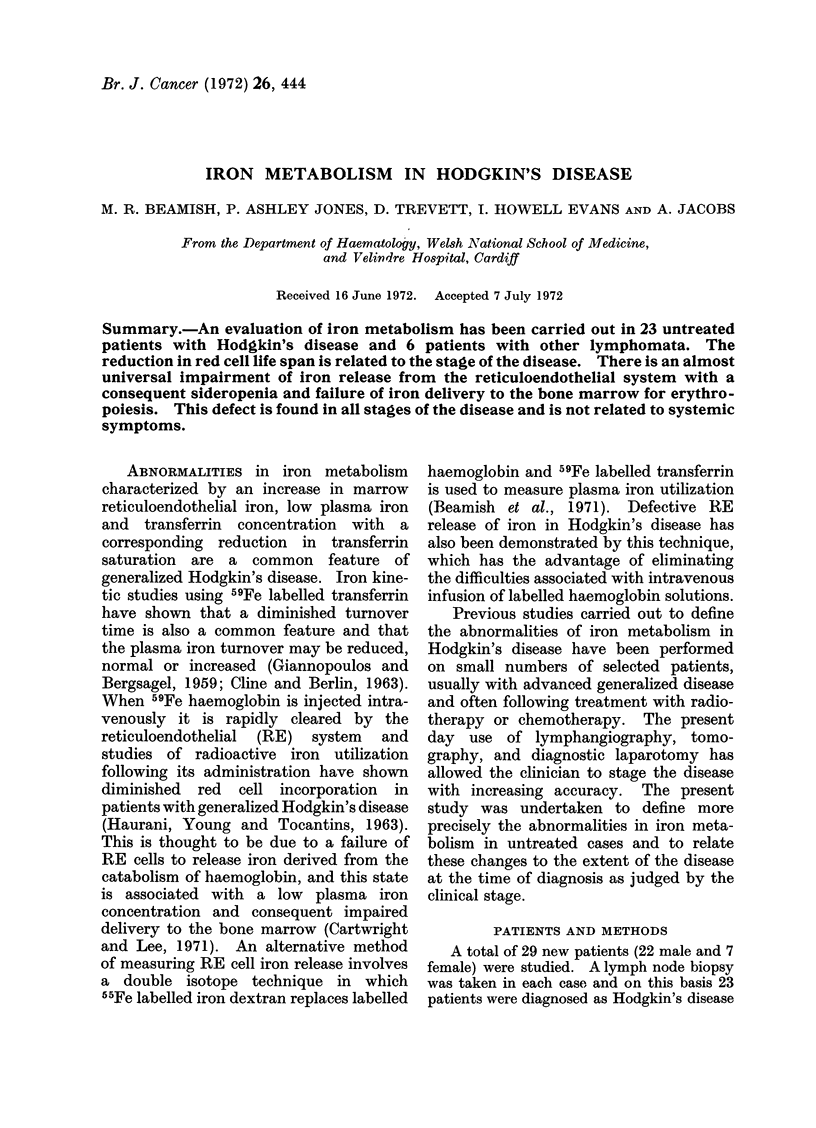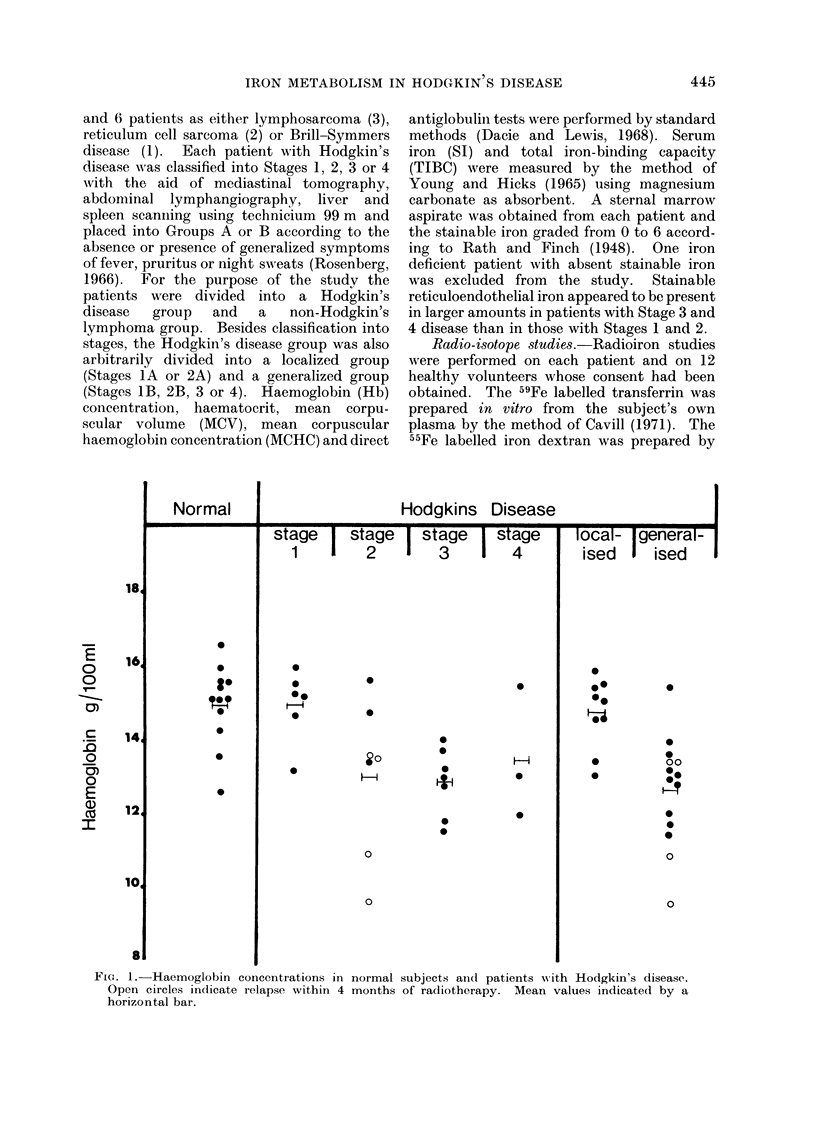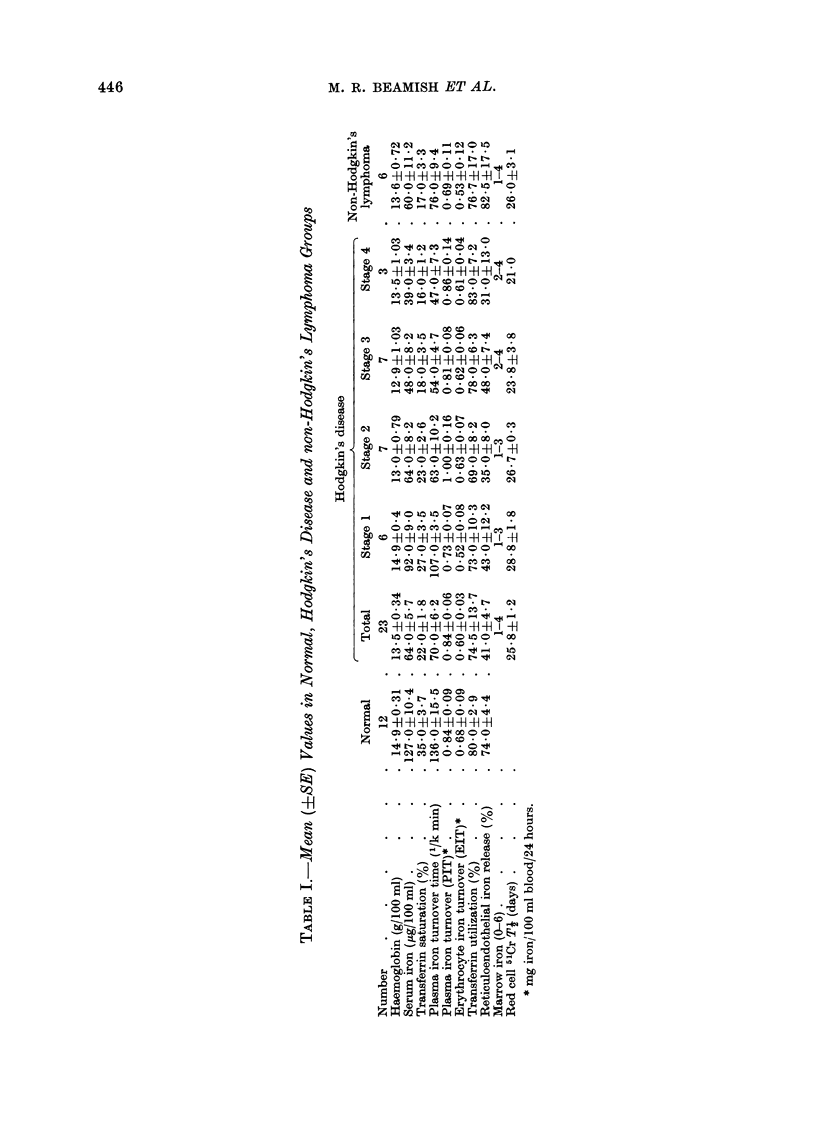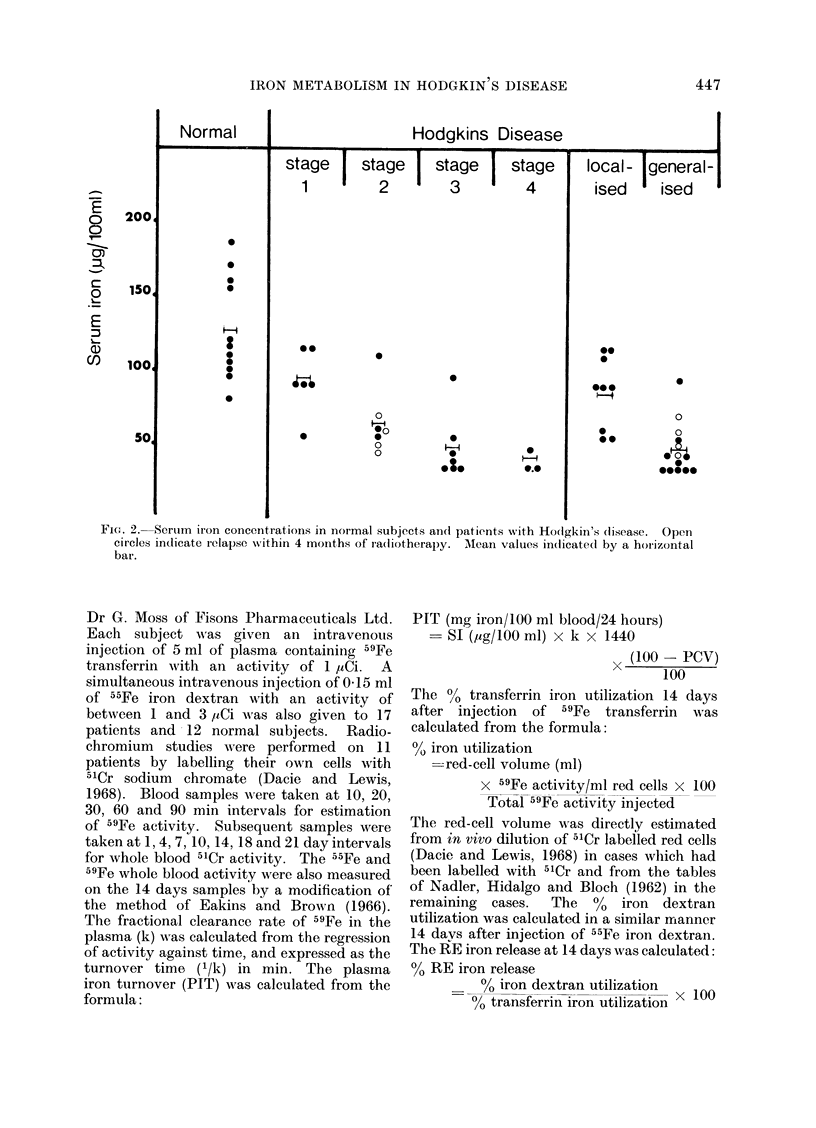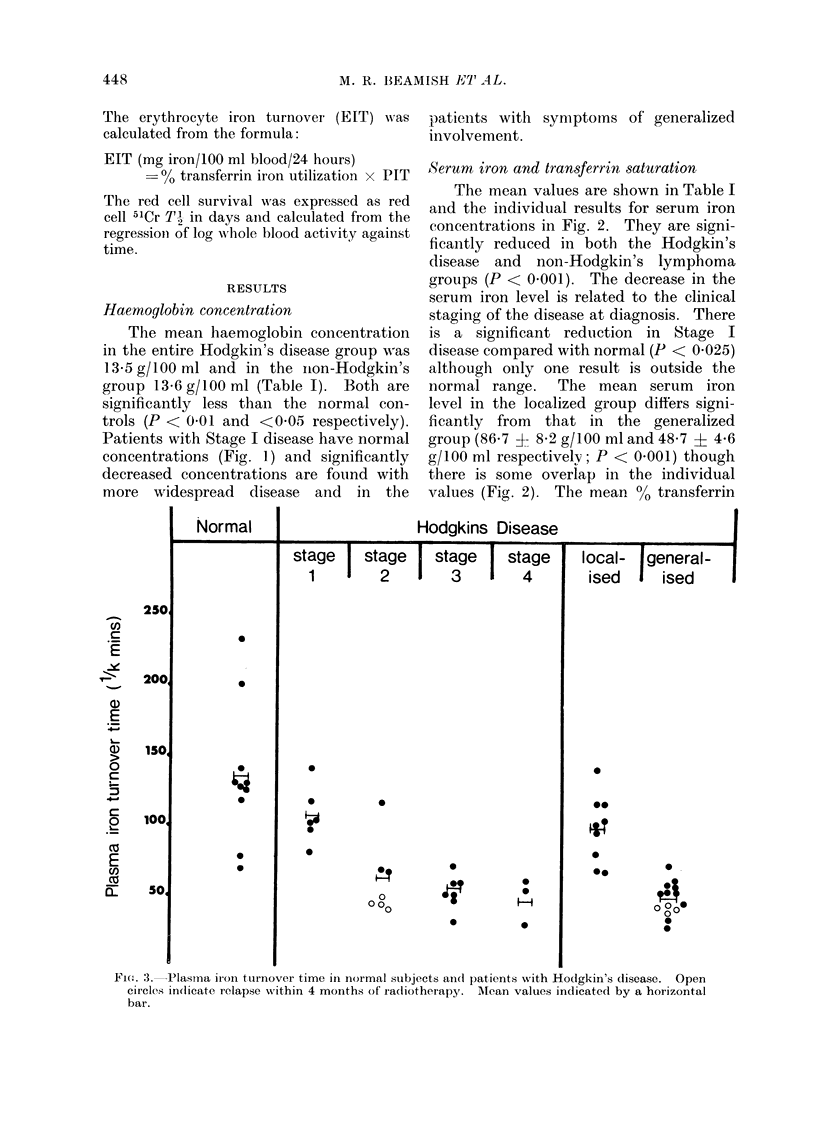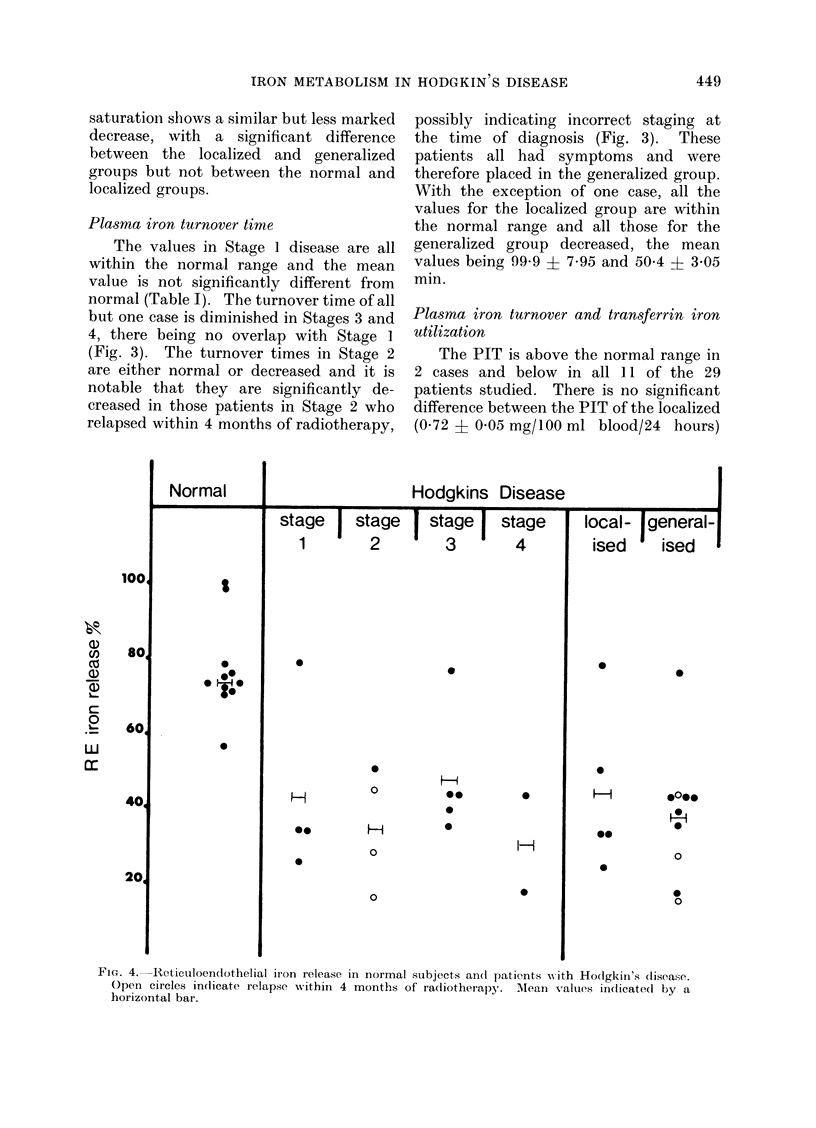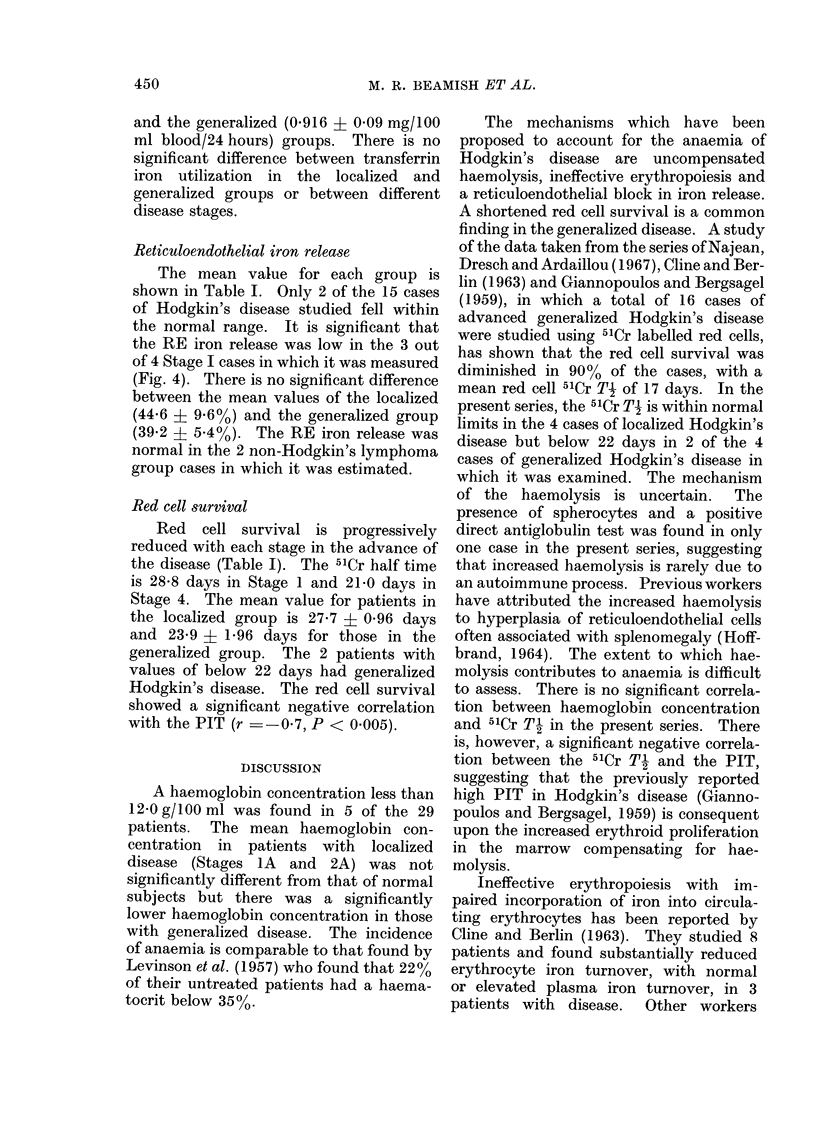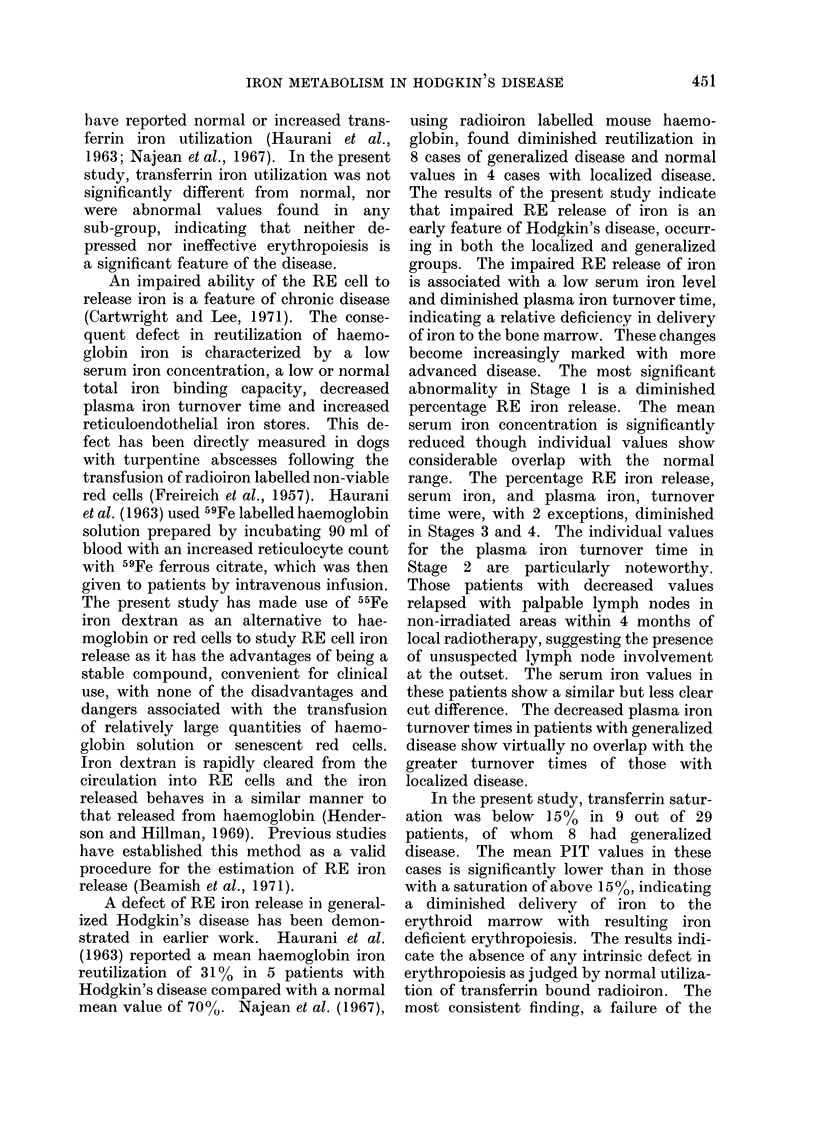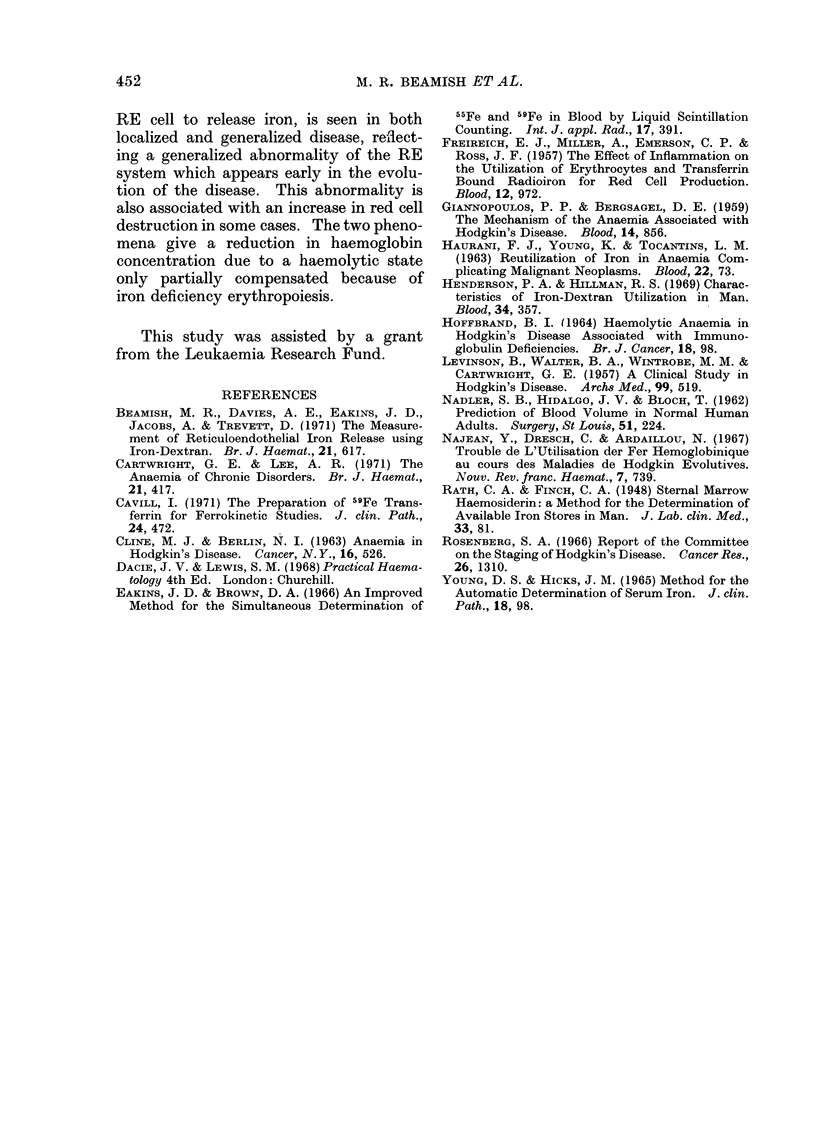# Iron Metabolism in Hodgkin's Disease

**DOI:** 10.1038/bjc.1972.61

**Published:** 1972-12

**Authors:** M. R. Beamish, P. Ashley Jones, D. Trevett, I. Howell Evans, A. Jacobs

## Abstract

An evaluation of iron metabolism has been carried out in 23 untreated patients with Hodgkin's disease and 6 patients with other lymphomata. The reduction in red cell life span is related to the stage of the disease. There is an almost universal impairment of iron release from the reticuloendothelial system with a consequent sideropenia and failure of iron delivery to the bone marrow for erythropoiesis. This defect is found in all stages of the disease and is not related to systemic symptoms.


*Br. J. Cancer* (1972) **26**, 444

### IRON METABOLISM IN HODGKIN'S DISEASE

M. R. BEAMISH, P. ASHLEY JONES, D. TREVETT, I. HOWELL EVANS AND A. JACOBS

*From the Department of Haematology, Welsh National School of Medicine, and Velindre Hospital, Cardiff*

Received 16 June 1972. Accepted 7 July 1972

**Summary.—An evaluation of iron metabolism has been carried out in 23 untreated patients with Hodgkin's disease and 6 patients with other lymphomata. The reduction in red cell life span is related to the stage of the disease. There is an almost universal impairment of iron release from the reticuloendothelial system with a consequent sideropenia and failure of iron delivery to the bone marrow for erythropoiesis. This defect is found in all stages of the disease and is not related to systemic symptoms.**

ABNORMALITIES in iron metabolism characterized by an increase in marrow reticuloendothelial iron, low plasma iron and transferrin concentration with a corresponding reduction in transferrin saturation are a common feature of generalized Hodgkin's disease. Iron kinetic studies using $^{59}$Fe labelled transferrin have shown that a diminished turnover time is also a common feature and that the plasma iron turnover may be reduced, normal or increased (Giannopoulos and Bergsagel, 1959; Cline and Berlin, 1963). When $^{59}$Fe haemoglobin is injected intravenously it is rapidly cleared by the reticuloendothelial (RE) system and studies of radioactive iron utilization following its administration have shown diminished red cell incorporation in patients with generalized Hodgkin's disease (Haurani, Young and Tocantins, 1963). This is thought to be due to a failure of RE cells to release iron derived from the catabolism of haemoglobin, and this state is associated with a low plasma iron concentration and consequent impaired delivery to the bone marrow (Cartwright and Lee, 1971). An alternative method of measuring RE cell iron release involves a double isotope technique in which $^{55}$Fe labelled iron dextran replaces labelled haemoglobin and $^{59}$Fe labelled transferrin is used to measure plasma iron utilization (Beamish *et al.*, 1971). Defective RE release of iron in Hodgkin's disease has also been demonstrated by this technique, which has the advantage of eliminating the difficulties associated with intravenous infusion of labelled haemoglobin solutions.

Previous studies carried out to define the abnormalities of iron metabolism in Hodgkin's disease have been performed on small numbers of selected patients, usually with advanced generalized disease and often following treatment with radiotherapy or chemotherapy. The present day use of lymphangiography, tomography, and diagnostic laparotomy has allowed the clinician to stage the disease with increasing accuracy. The present study was undertaken to define more precisely the abnormalities in iron metabolism in untreated cases and to relate these changes to the extent of the disease at the time of diagnosis as judged by the clinical stage.

### PATIENTS AND METHODS

A total of 29 new patients (22 male and 7 female) were studied. A lymph node biopsy was taken in each case and on this basis 23 patients were diagnosed as Hodgkin's disease and 6 patients as either lymphosarcoma (3), reticulum cell sarcoma (2) or Brill–Symmers disease (1). Each patient with Hodgkin's disease was classified into Stages 1, 2, 3 or 4 with the aid of mediastinal tomography, abdominal lymphangiography, liver and spleen scanning using technicium 99 m and placed into Groups A or B according to the absence or presence of generalized symptoms of fever, pruritus or night sweats (Rosenberg, 1966). For the purpose of the study the patients were divided into a Hodgkin's disease group and a non-Hodgkin's lymphoma group. Besides classification into stages, the Hodgkin's disease group was also arbitrarily divided into a localized group (Stages 1A or 2A) and a generalized group (Stages 1B, 2B, 3 or 4). Haemoglobin (Hb) concentration, haematocrit, mean corpuscular volume (MCV), mean corpuscular haemoglobin concentration (MCHC) and direct antiglobulin tests were performed by standard methods (Dacie and Lewis, 1968). Serum iron (SI) and total iron-binding capacity (TIBC) were measured by the method of Young and Hicks (1965) using magnesium carbonate as absorbent. A sternal marrow aspirate was obtained from each patient and the stainable iron graded from 0 to 6 according to Rath and Finch (1948). One iron deficient patient with absent stainable iron was excluded from the study. Stainable reticuloendothelial iron appeared to be present in larger amounts in patients with Stage 3 and 4 disease than in those with Stages 1 and 2.

*Radio-isotope studies.*—Radioiron studies were performed on each patient and on 12 healthy volunteers whose consent had been obtained. The $^{59}$Fe labelled transferrin was prepared *in vitro* from the subject's own plasma by the method of Cavill (1971). The $^{55}$Fe labelled iron dextran was prepared by

FIG. 1.—Haemoglobin concentrations in normal subjects and patients with Hodgkin's disease. Open circles indicate relapse within 4 months of radiotherapy. Mean values indicated by a horizontal bar.

TABLE I.—*Mean* (±*SE*) *Values in Normal, Hodgkin's Disease and non-Hodgkin's Lymphoma Groups*

| | | Hodgkin's disease | | | | | |
|---|---|---|---|---|---|---|---|
| | Normal | Total | Stage 1 | Stage 2 | Stage 3 | Stage 4 | Non-Hodgkin's lymphoma |
| Number | 12 | 23 | 6 | 7 | 7 | 3 | 6 |
| Haemoglobin (g/100 ml) | 14·9±0·31 | 13·5±0·34 | 14·9±0·4 | 13·0±0·79 | 12·9±1·03 | 13·5±1·03 | 13·6±0·72 |
| Serum iron (μg/100 ml) | 127·0±10·4 | 64·0±5·7 | 92·0±9·0 | 64·0±8·2 | 48·0±8·2 | 39·0±3·4 | 60·0±11·2 |
| Transferrin saturation (%) | 35·0±3·7 | 22·0±1·8 | 27·0±3·5 | 23·0±2·6 | 18·0±3·5 | 16·0±1·2 | 17·0±3·3 |
| Plasma iron turnover time ($^1$/k min) | 136·0±15·5 | 70·0±6·2 | 107·0±3·5 | 63·0±10·2 | 54·0±4·7 | 47·0±7·3 | 76·0±9·4 |
| Plasma iron turnover (PIT)* | 0·84±0·09 | 0·84±0·06 | 0·73±0·07 | 1·00±0·16 | 0·81±0·08 | 0·86±0·14 | 0·69±0·11 |
| Erythrocyte iron turnover (EIT)* | 0·68±0·09 | 0·60±0·03 | 0·52±0·08 | 0·63±0·07 | 0·62±0·06 | 0·61±0·04 | 0·53±0·12 |
| Transferrin utilization (%) | 80·0±2·9 | 74·5±13·7 | 73·0±10·3 | 69·0±8·2 | 78·0±6·3 | 83·0±7·2 | 76·7±17·0 |
| Reticuloendothelial iron release (%) | 74·0±4·4 | 41·0±4·7 | 43·0±12·2 | 35·0±8·0 | 48·0±7·4 | 31·0±13·0 | 82·5±17·5 |
| Marrow iron (0–6) | | 1–4 | 1–3 | 1–3 | 2–4 | 2–4 | 1–4 |
| Red cell $^{51}Cr$ $T\frac{1}{2}$ (days) | | 25·8±1·2 | 28·8±1·8 | 26·7±0·3 | 23·8±3·8 | 21·0 | 26·0±3·1 |

* mg iron/100 ml blood/24 hours.

Fig. 2.—Serum iron concentrations in normal subjects and patients with Hodgkin's disease. Open circles indicate relapse within 4 months of radiotherapy. Mean values indicated by a horizontal bar.

Dr G. Moss of Fisons Pharmaceuticals Ltd. Each subject was given an intravenous injection of 5 ml of plasma containing $^{59}Fe$ transferrin with an activity of 1 $\mu$Ci. A simultaneous intravenous injection of 0·15 ml of $^{55}Fe$ iron dextran with an activity of between 1 and 3 $\mu$Ci was also given to 17 patients and 12 normal subjects. Radiochromium studies were performed on 11 patients by labelling their own cells with $^{51}Cr$ sodium chromate (Dacie and Lewis, 1968). Blood samples were taken at 10, 20, 30, 60 and 90 min intervals for estimation of $^{59}Fe$ activity. Subsequent samples were taken at 1, 4, 7, 10, 14, 18 and 21 day intervals for whole blood $^{51}Cr$ activity. The $^{55}Fe$ and $^{59}Fe$ whole blood activity were also measured on the 14 days samples by a modification of the method of Eakins and Brown (1966). The fractional clearance rate of $^{59}Fe$ in the plasma (k) was calculated from the regression of activity against time, and expressed as the turnover time ($^1/k$) in min. The plasma iron turnover (PIT) was calculated from the formula:

$$\text{PIT (mg iron/100 ml blood/24 hours)} = \text{SI } (\mu g/100 \text{ ml}) \times k \times 1440 \times \frac{(100 - \text{PCV})}{100}$$

The % transferrin iron utilization 14 days after injection of $^{59}Fe$ transferrin was calculated from the formula:

$$\% \text{ iron utilization} = \text{red-cell volume (ml)} \times \frac{{}^{59}\text{Fe activity/ml red cells} \times 100}{\text{Total } {}^{59}\text{Fe activity injected}}$$

The red-cell volume was directly estimated from *in vivo* dilution of $^{51}Cr$ labelled red cells (Dacie and Lewis, 1968) in cases which had been labelled with $^{51}Cr$ and from the tables of Nadler, Hidalgo and Bloch (1962) in the remaining cases. The % iron dextran utilization was calculated in a similar manner 14 days after injection of $^{55}Fe$ iron dextran. The RE iron release at 14 days was calculated:

$$\% \text{ RE iron release} = \frac{\% \text{ iron dextran utilization}}{\% \text{ transferrin iron utilization}} \times 100$$

The erythrocyte iron turnover (EIT) was calculated from the formula:

EIT (mg iron/100 ml blood/24 hours)
$= \%$ transferrin iron utilization $\times$ PIT

The red cell survival was expressed as red cell $^{51}$Cr $T\frac{1}{2}$ in days and calculated from the regression of log whole blood activity against time.

### RESULTS

### *Haemoglobin concentration*

The mean haemoglobin concentration in the entire Hodgkin's disease group was 13·5 g/100 ml and in the non-Hodgkin's group 13·6 g/100 ml (Table I). Both are significantly less than the normal controls ($P < 0{\cdot}01$ and $<0{\cdot}05$ respectively). Patients with Stage I disease have normal concentrations (Fig. 1) and significantly decreased concentrations are found with more widespread disease and in the patients with symptoms of generalized involvement.

### *Serum iron and transferrin saturation*

The mean values are shown in Table I and the individual results for serum iron concentrations in Fig. 2. They are significantly reduced in both the Hodgkin's disease and non-Hodgkin's lymphoma groups ($P < 0{\cdot}001$). The decrease in the serum iron level is related to the clinical staging of the disease at diagnosis. There is a significant reduction in Stage I disease compared with normal ($P < 0{\cdot}025$) although only one result is outside the normal range. The mean serum iron level in the localized group differs significantly from that in the generalized group (86·7 ± 8·2 g/100 ml and 48·7 ± 4·6 g/100 ml respectively; $P < 0{\cdot}001$) though there is some overlap in the individual values (Fig. 2). The mean % transferrin

FIG. 3.—Plasma iron turnover time in normal subjects and patients with Hodgkin's disease. Open circles indicate relapse within 4 months of radiotherapy. Mean values indicated by a horizontal bar.

saturation shows a similar but less marked decrease, with a significant difference between the localized and generalized groups but not between the normal and localized groups.

*Plasma iron turnover time*

The values in Stage 1 disease are all within the normal range and the mean value is not significantly different from normal (Table I). The turnover time of all but one case is diminished in Stages 3 and 4, there being no overlap with Stage 1 (Fig. 3). The turnover times in Stage 2 are either normal or decreased and it is notable that they are significantly decreased in those patients in Stage 2 who relapsed within 4 months of radiotherapy, possibly indicating incorrect staging at the time of diagnosis (Fig. 3). These patients all had symptoms and were therefore placed in the generalized group. With the exception of one case, all the values for the localized group are within the normal range and all those for the generalized group decreased, the mean values being 99·9 ± 7·95 and 50·4 ± 3·05 min.

*Plasma iron turnover and transferrin iron utilization*

The PIT is above the normal range in 2 cases and below in all 11 of the 29 patients studied. There is no significant difference between the PIT of the localized (0·72 ± 0·05 mg/100 ml blood/24 hours)

FIG. 4.—Reticuloendothelial iron release in normal subjects and patients with Hodgkin's disease. Open circles indicate relapse within 4 months of radiotherapy. Mean values indicated by a horizontal bar.

and the generalized ($0{\cdot}916 \pm 0{\cdot}09$ mg/100 ml blood/24 hours) groups. There is no significant difference between transferrin iron utilization in the localized and generalized groups or between different disease stages.

*Reticuloendothelial iron release*

The mean value for each group is shown in Table I. Only 2 of the 15 cases of Hodgkin's disease studied fell within the normal range. It is significant that the RE iron release was low in the 3 out of 4 Stage I cases in which it was measured (Fig. 4). There is no significant difference between the mean values of the localized ($44{\cdot}6 \pm 9{\cdot}6\%$) and the generalized group ($39{\cdot}2 \pm 5{\cdot}4\%$). The RE iron release was normal in the 2 non-Hodgkin's lymphoma group cases in which it was estimated.

*Red cell survival*

Red cell survival is progressively reduced with each stage in the advance of the disease (Table I). The $^{51}$Cr half time is 28·8 days in Stage 1 and 21·0 days in Stage 4. The mean value for patients in the localized group is $27{\cdot}7 \pm 0{\cdot}96$ days and $23{\cdot}9 \pm 1{\cdot}96$ days for those in the generalized group. The 2 patients with values of below 22 days had generalized Hodgkin's disease. The red cell survival showed a significant negative correlation with the PIT ($r = -0{\cdot}7$, $P < 0{\cdot}005$).

DISCUSSION

A haemoglobin concentration less than 12·0 g/100 ml was found in 5 of the 29 patients. The mean haemoglobin concentration in patients with localized disease (Stages 1A and 2A) was not significantly different from that of normal subjects but there was a significantly lower haemoglobin concentration in those with generalized disease. The incidence of anaemia is comparable to that found by Levinson *et al.* (1957) who found that 22% of their untreated patients had a haematocrit below 35%.

The mechanisms which have been proposed to account for the anaemia of Hodgkin's disease are uncompensated haemolysis, ineffective erythropoiesis and a reticuloendothelial block in iron release. A shortened red cell survival is a common finding in the generalized disease. A study of the data taken from the series of Najean, Dresch and Ardaillou (1967), Cline and Berlin (1963) and Giannopoulos and Bergsagel (1959), in which a total of 16 cases of advanced generalized Hodgkin's disease were studied using $^{51}$Cr labelled red cells, has shown that the red cell survival was diminished in 90% of the cases, with a mean red cell $^{51}$Cr $T\frac{1}{2}$ of 17 days. In the present series, the $^{51}$Cr $T\frac{1}{2}$ is within normal limits in the 4 cases of localized Hodgkin's disease but below 22 days in 2 of the 4 cases of generalized Hodgkin's disease in which it was examined. The mechanism of the haemolysis is uncertain. The presence of spherocytes and a positive direct antiglobulin test was found in only one case in the present series, suggesting that increased haemolysis is rarely due to an autoimmune process. Previous workers have attributed the increased haemolysis to hyperplasia of reticuloendothelial cells often associated with splenomegaly (Hoffbrand, 1964). The extent to which haemolysis contributes to anaemia is difficult to assess. There is no significant correlation between haemoglobin concentration and $^{51}$Cr $T\frac{1}{2}$ in the present series. There is, however, a significant negative correlation between the $^{51}$Cr $T\frac{1}{2}$ and the PIT, suggesting that the previously reported high PIT in Hodgkin's disease (Giannopoulos and Bergsagel, 1959) is consequent upon the increased erythroid proliferation in the marrow compensating for haemolysis.

Ineffective erythropoiesis with impaired incorporation of iron into circulating erythrocytes has been reported by Cline and Berlin (1963). They studied 8 patients and found substantially reduced erythrocyte iron turnover, with normal or elevated plasma iron turnover, in 3 patients with disease. Other workers have reported normal or increased transferrin iron utilization (Haurani *et al.*, 1963; Najean *et al.*, 1967). In the present study, transferrin iron utilization was not significantly different from normal, nor were abnormal values found in any sub-group, indicating that neither depressed nor ineffective erythropoiesis is a significant feature of the disease.

An impaired ability of the RE cell to release iron is a feature of chronic disease (Cartwright and Lee, 1971). The consequent defect in reutilization of haemoglobin iron is characterized by a low serum iron concentration, a low or normal total iron binding capacity, decreased plasma iron turnover time and increased reticuloendothelial iron stores. This defect has been directly measured in dogs with turpentine abscesses following the transfusion of radioiron labelled non-viable red cells (Freireich *et al.*, 1957). Haurani *et al.* (1963) used $^{59}$Fe labelled haemoglobin solution prepared by incubating 90 ml of blood with an increased reticulocyte count with $^{59}$Fe ferrous citrate, which was then given to patients by intravenous infusion. The present study has made use of $^{55}$Fe iron dextran as an alternative to haemoglobin or red cells to study RE cell iron release as it has the advantages of being a stable compound, convenient for clinical use, with none of the disadvantages and dangers associated with the transfusion of relatively large quantities of haemoglobin solution or senescent red cells. Iron dextran is rapidly cleared from the circulation into RE cells and the iron released behaves in a similar manner to that released from haemoglobin (Henderson and Hillman, 1969). Previous studies have established this method as a valid procedure for the estimation of RE iron release (Beamish *et al.*, 1971).

A defect of RE iron release in generalized Hodgkin's disease has been demonstrated in earlier work. Haurani *et al.* (1963) reported a mean haemoglobin iron reutilization of 31% in 5 patients with Hodgkin's disease compared with a normal mean value of 70%. Najean *et al.* (1967), using radioiron labelled mouse haemoglobin, found diminished reutilization in 8 cases of generalized disease and normal values in 4 cases with localized disease. The results of the present study indicate that impaired RE release of iron is an early feature of Hodgkin's disease, occurring in both the localized and generalized groups. The impaired RE release of iron is associated with a low serum iron level and diminished plasma iron turnover time, indicating a relative deficiency in delivery of iron to the bone marrow. These changes become increasingly marked with more advanced disease. The most significant abnormality in Stage 1 is a diminished percentage RE iron release. The mean serum iron concentration is significantly reduced though individual values show considerable overlap with the normal range. The percentage RE iron release, serum iron, and plasma iron, turnover time were, with 2 exceptions, diminished in Stages 3 and 4. The individual values for the plasma iron turnover time in Stage 2 are particularly noteworthy. Those patients with decreased values relapsed with palpable lymph nodes in non-irradiated areas within 4 months of local radiotherapy, suggesting the presence of unsuspected lymph node involvement at the outset. The serum iron values in these patients show a similar but less clear cut difference. The decreased plasma iron turnover times in patients with generalized disease show virtually no overlap with the greater turnover times of those with localized disease.

In the present study, transferrin saturation was below 15% in 9 out of 29 patients, of whom 8 had generalized disease. The mean PIT values in these cases is significantly lower than in those with a saturation of above 15%, indicating a diminished delivery of iron to the erythroid marrow with resulting iron deficient erythropoiesis. The results indicate the absence of any intrinsic defect in erythropoiesis as judged by normal utilization of transferrin bound radioiron. The most consistent finding, a failure of the

RE cell to release iron, is seen in both localized and generalized disease, reflecting a generalized abnormality of the RE system which appears early in the evolution of the disease. This abnormality is also associated with an increase in red cell destruction in some cases. The two phenomena give a reduction in haemoglobin concentration due to a haemolytic state only partially compensated because of iron deficiency erythropoiesis.

This study was assisted by a grant from the Leukaemia Research Fund.

### REFERENCES

BEAMISH, M. R., DAVIES, A. E., EAKINS, J. D., JACOBS, A. & TREVETT, D. (1971) The Measurement of Reticuloendothelial Iron Release using Iron-Dextran. *Br. J. Haemat.*, **21**, 617.

CARTWRIGHT, G. E. & LEE, A. R. (1971) The Anaemia of Chronic Disorders. *Br. J. Haemat.*, **21**, 417.

CAVILL, I. (1971) The Preparation of $^{59}$Fe Transferrin for Ferrokinetic Studies. *J. clin. Path.*, **24**, 472.

CLINE, M. J. & BERLIN, N. I. (1963) Anaemia in Hodgkin's Disease. *Cancer, N.Y.*, **16**, 526.

DACIE, J. V. & LEWIS, S. M. (1968) *Practical Haematology* 4th Ed. London: Churchill.

EAKINS, J. D. & BROWN, D. A. (1966) An Improved Method for the Simultaneous Determination of $^{55}$Fe and $^{59}$Fe in Blood by Liquid Scintillation Counting. *Int. J. appl. Rad.*, **17**, 391.

FREIREICH, E. J., MILLER, A., EMERSON, C. P. & ROSS, J. F. (1957) The Effect of Inflammation on the Utilization of Erythrocytes and Transferrin Bound Radioiron for Red Cell Production. *Blood*, **12**, 972.

GIANNOPOULOS, P. P. & BERGSAGEL, D. E. (1959) The Mechanism of the Anaemia Associated with Hodgkin's Disease. *Blood*, **14**, 856.

HAURANI, F. J., YOUNG, K. & TOCANTINS, L. M. (1963) Reutilization of Iron in Anaemia Complicating Malignant Neoplasms. *Blood*, **22**, 73.

HENDERSON, P. A. & HILLMAN, R. S. (1969) Characteristics of Iron-Dextran Utilization in Man. *Blood*, **34**, 357.

HOFFBRAND, B. I. (1964) Haemolytic Anaemia in Hodgkin's Disease Associated with Immunoglobulin Deficiencies. *Br. J. Cancer*, **18**, 98.

LEVINSON, B., WALTER, B. A., WINTROBE, M. M. & CARTWRIGHT, G. E. (1957) A Clinical Study in Hodgkin's Disease. *Archs Med.*, **99**, 519.

NADLER, S. B., HIDALGO, J. V. & BLOCH, T. (1962) Prediction of Blood Volume in Normal Human Adults. *Surgery, St Louis*, **51**, 224.

NAJEAN, Y., DRESCH, C. & ARDAILLOU, N. (1967) Trouble de L'Utilisation der Fer Hemoglobinique au cours des Maladies de Hodgkin Evolutives. *Nouv. Rev. franc. Haemat.*, **7**, 739.

RATH, C. A. & FINCH, C. A. (1948) Sternal Marrow Haemosiderin: a Method for the Determination of Available Iron Stores in Man. *J. Lab. clin. Med.*, **33**, 81.

ROSENBERG, S. A. (1966) Report of the Committee on the Staging of Hodgkin's Disease. *Cancer Res.*, **26**, 1310.

YOUNG, D. S. & HICKS, J. M. (1965) Method for the Automatic Determination of Serum Iron. *J. clin. Path.*, **18**, 98.